# Inborn Errors of the Immune System Associated With Atopy

**DOI:** 10.3389/fimmu.2022.860821

**Published:** 2022-04-27

**Authors:** Ryan W. Nelson, Raif S. Geha, Douglas R. McDonald

**Affiliations:** Division of Immunology, Boston Children’s Hospital, Harvard Medical School, Boston, MA, United States

**Keywords:** atopy, inborn errors of immunity (IEI), barrier function, cytoskeletal, TCR, cytokine, T cell

## Abstract

Atopic disorders, including atopic dermatitis, food and environmental allergies, and asthma, are increasingly prevalent diseases. Atopic disorders are often associated with eosinophilia, driven by T helper type 2 (Th2) immune responses, and triggered by disrupted barrier function leading to abnormal immune priming in a susceptible host. Immune deficiencies, in contrast, occur with a significantly lower incidence, but are associated with greater morbidity and mortality. A subset of atopic disorders with eosinophilia and elevated IgE are associated with monogenic inborn errors of immunity (IEI). In this review, we discuss current knowledge of IEI that are associated with atopy and the lessons these immunologic disorders provide regarding the fundamental mechanisms that regulate type 2 immunity in humans. We also discuss further mechanistic insights provided by animal models.

## Introduction

Atopic diseases are common and result from dysregulated type 2 immunity driven by T helper type 2 (Th2) cells. Th2 cells are protective to the host when coordinated in response to specific helminth pathogens or toxins but can also induce tissue pathology when over-active or misdirected toward innocuous stimuli. Th2 effector functions are mediated primarily through secretion of pro-inflammatory cytokines including interleukin (IL)-4, IL-5, and IL-13 to activate and recruit effector cells such as mast cells and eosinophils. A fraction of Th2 cells home to peripheral tissues where they permanently reside as tissue-resident memory cells (Th2-Trm) (1–6). T follicular helper (Tfh) cells formed in the context of type 2 immune responses reside primarily in secondary lymphoid tissues and promote B-cell class switching to immunoglobulin E (IgE) production (7).

There is polygenic heritable risk for development of atopic diseases, and the increasing prevalence of these disorders also points toward changing environmental factors significantly influencing susceptibility. Living on a farm is a strong protective factor against the development of asthma (8), and childhood microbial exposures measured by home endotoxin levels have demonstrated a similar inverse relationship with asthma and environmental allergy prevalence (9). Specific gene–environment interactions have been shown to affect atopic disease activity in humans. For example, individuals homozygous for the Q576R risk allele in the IL-4 receptor alpha chain gene (*IL4RA*) demonstrated increased asthma symptoms when exposed to higher school endotoxin levels, whereas individuals that did not harbor this allelic variant showed protective benefit (10). However, complex gene plus environment interactions that result in atopic dermatitis, allergies, asthma, and other immune-mediated diseases in humans are difficult to study and recapitulate in animal models. This is in part due to the large sample sizes needed for controlled human studies related to polygenic diseases and imperfect methods for sampling and quantifying environmental variables (11).

Mouse models have been instrumental in elucidating immune pathways of atopic disease given the advantage of controlled genetics and allergic sensitization protocols. This work has solidified the principle that dysregulated type 2 immune responses can result from both immune cell-intrinsic and -extrinsic defects. Initially described in wild-type BALB/c mice, epicutaneous exposure to foreign protein antigens through tape-stripped skin elicits type 2 inflammation resembling atopic dermatitis (12). The phenotype in mice becomes much more pronounced with gene-targeted alterations that directly promote type 2 inflammation or repress mechanisms critical for normal immune homeostasis. Thus, either disrupted barrier function or immune dysregulation appear central to atopic diatheses, and monogenic disorders in humans provide additional insights that will be reviewed here.

Primary immunodeficiencies (PIDs) have significantly contributed to our understanding of human immunity to infection. More recently, monogenic variants associated with early and severe autoinflammation, autoimmunity, and atopic disease have been described. Inborn errors of immunity (IEI) now encompass monogenic immune dysregulation disorders in addition to immune deficiencies. These comprise autoinflammatory conditions affecting the innate immune system and autoimmune and allergic disorders primarily affecting the adaptive immune system. At the time of this review, IEI with mutations in more than 400 genes have been reported (13), and this number continues to rise as awareness of IEI grows and genetic testing becomes more readily available. The shared and unique clinical phenotypes and immune pathways involved in these inborn errors offer opportunities to gain insight into the pathogenesis of immune dysregulation disorders.

Monogenic IEI associated with atopy, or primary atopic disorders (14, 15), have implicated interrelated pathways involving the actin cytoskeleton and immunological synapse formation, aberrant T-cell receptor (TCR) signaling, cytokine signaling pathways, T-cell repertoire diversity, balance of regulatory T cells (Tregs) and effector T cells, and innate immune cell effector mechanisms as critical factors influencing type 2 inflammation and atopic disease in humans. Contextualized with mechanistic paradigms developed largely in experimental animal models, IEI may also provide significant insight into common atopic disease mechanisms and lead to improved prevention and treatment strategies.

## Disorders of Barrier Function

Gene defects associated with skin barrier function have been associated with early and severe atopic dermatitis and allergen sensitization. While not immunodeficiencies *per se* as they involve non-hematopoietic cells, these mutations may increase risk of infections secondary to disrupted barrier to microbial penetration and to the inhibitory effect of cutaneously expressed Th2 cytokines on the production of antimicrobial peptides by keratinocytes. Skin permeability also allows entry of allergens and other foreign antigens, toxins, and irritants. Subsequent production of pro-inflammatory type 2 cytokines leads to a feed-forward loop locally in the skin and also affects immune cells residing in other peripheral tissues. Clinically, atopic dermatitis has long been appreciated as the primary manifestation in individuals with multiple atopic diseases. The natural history of atopic dermatitis early in life transitioning to later development of food allergies, asthma, and allergic rhinitis is frequently described as the “atopic march” (16).

**Figure 1:**
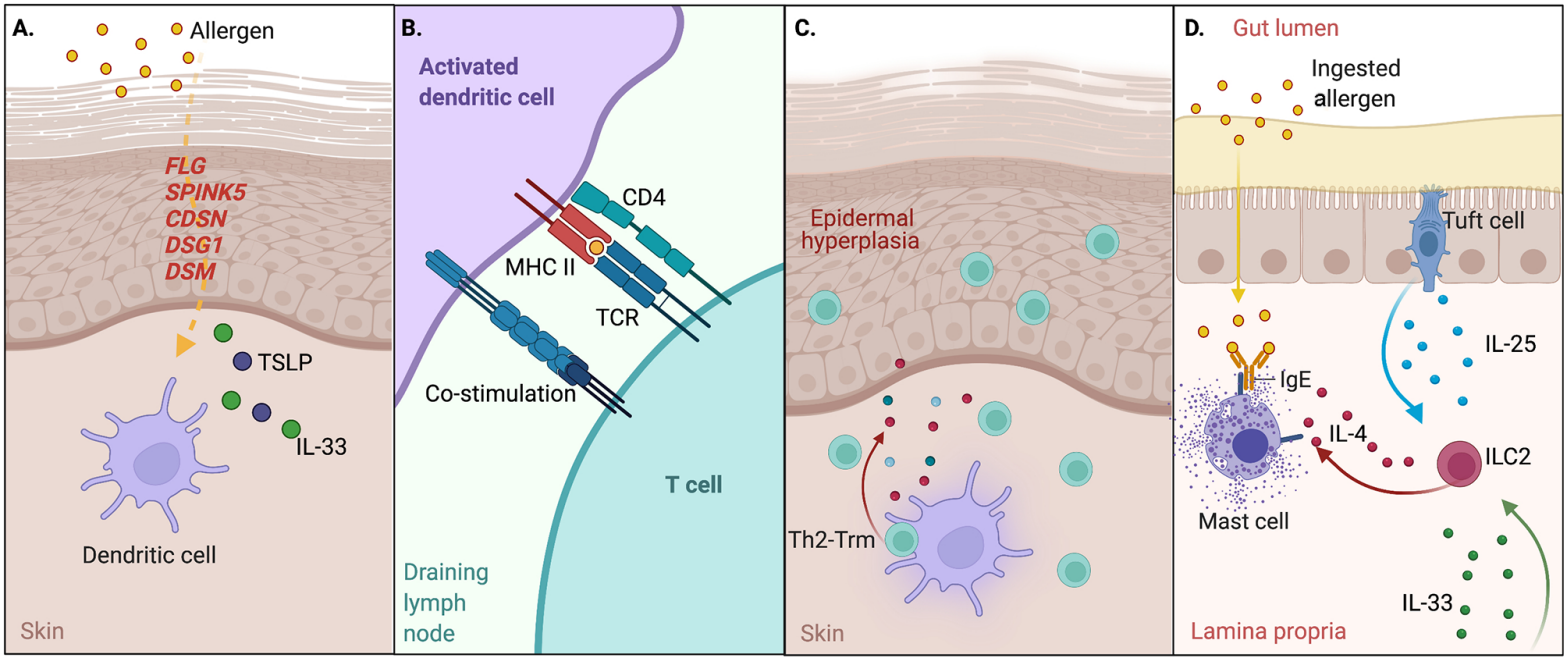
Disrupted barrier function and the atopic march. **(A)** Disrupted skin barrier through primary disorders of the skin or external factors enable allergen entry; uptake by dendritic cells in the presence of alarmin signals such as TSLP and IL-33 promotes dendritic cell maturation and migration to the draining LN. Genes noted in red indicate monogenic IEI affecting this pathway. **(B)** Dendritic cells present allergen-derived peptide:MHCII to naive CD4^+^ T cells to induce Th2 effector cells and Tfh cells that drive IgE responses to allergen. **(C)** A portion of Th2 cells traffic back to skin and permanently reside as tissue-resident memory cells to drive local inflammation through release of type 2 cytokines such as IL-4, -5, and -13 upon re-encountering allergen. **(D)** Systemic IL-33 from keratinocytes and IL-25 from intestinal tuft cells synergize to activate ILC2s, which in turn produce IL-4 and activate mast cell degranulation (anaphylaxis) in response to oral allergen exposure.

Ichthyosis vulgaris is an inherited disorder of skin keratinization presenting in the first months of life with skin scaling and palmar hyperlinearity, along with keratosis pilaris and atopic dermatitis. Originally discovered as homozygous and compound heterozygous individuals with ichthyosis vulgaris (17), loss-of-function (LOF) mutations in the *FLG* gene encoding filament aggregating protein (filaggrin) have been associated with early and severe atopy. Heterozygous carriers of *FLG* mutations are also at increased risk for atopic dermatitis, allergic rhinitis, asthma, and food allergy (18–20). Expressed as an inactive polymer by keratinocytes, profilaggrin is cleaved by serine proteases to generate filaggrin monomers, which are cross-linked to facilitate the impermeability of the stratum corneum, prevent water loss, and maintain the skin barrier (21). Lympho-epithelial Kazal-type-related inhibitor (LEKTI) is a serine protease inhibitor also expressed by keratinocytes. LOF mutations in *SPINK5*, the gene encoding LEKTI, lead to the Netherton syndrome with congenital ichthyosis, abnormal hair shafts, and severe atopy also extending beyond the skin (22–24). Loss of LEKTI function leads to increased protease activity affecting intercellular adhesions, which causes peeling of deeper layers of the skin (25). Disruption of keratinocyte cell junctions by mutations in genes encoding corneodesmosin (*CDSN*) (26, 27), desmoglein 1 (*DSG1*) (28), and desmoplakin (*DSP*) (29) involved in corneodesmosomes also cause skin barrier defects and severe atopy (**Figure 1A**).

Murine studies have also provided mechanistic explanations for the relationship between allergic sensitization through the skin and multiple atopic comorbidities affecting different tissues. Indeed, mice sensitized to chicken ovalbumin (OVA) or peanut through mechanically disrupted skin that are subsequently challenged to the same antigen in the airways or gut result in phenotypes resembling asthma, food allergen induced anaphylaxis, and eosinophilic esophagitis (12, 30, 31). Damaged keratinocytes express high levels of alarmins such as TSLP, IL-33, and IL-25 that promote type 2 inflammatory responses (32). Dendritic cells in the skin become activated in response to alarmin signals, migrate to skin draining lymph nodes, and present phagocytosed and processed foreign antigens to naïve CD4^+^ T cells in the form of peptides bound to MHCII (peptide:MHCII) to drive the Th2 differentiation program (33) and T-cell-dependent IgE production. Th2 cells subsequently traffic back to the skin where they reside as Th2-Trm and drive hallmark features of atopic dermatitis—inflammatory cell recruitment and epidermal hyperplasia. Recent studies also suggest that timing may be important for skin sensitization, as early life skin inflammation in mice enables a Th2-Trm niche with stromal cells in the skin (34). In the model of food allergy, systemic IL-33 produced by damaged keratinocytes synergizes with IL-25 produced by intestinal tuft cells (35) to promote activation and expansion of ILC2s in the gut, which in turn produce IL-4 locally to activate and expand mast cells to cause anaphylaxis following oral rechallenge with allergen (30) (**Figures 1B–D**).

## Cytoskeletal Abnormalities

**Figure 2:**
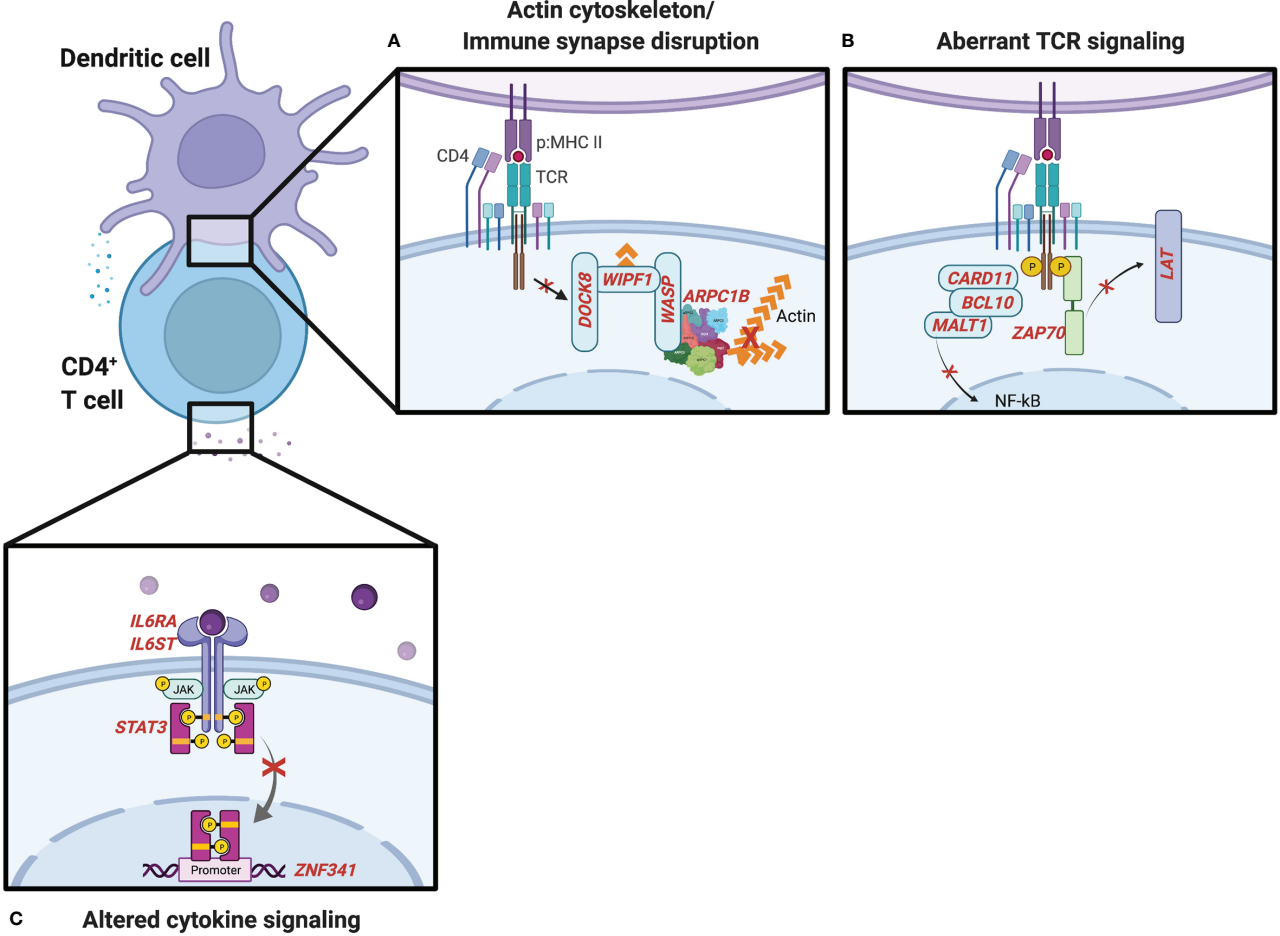
IEI influencing CD4^+^ T-cell activation and differentiation. **(A)** Genes noted in red involved in the actin cytoskeleton and formation of the immunological synapse. **(B)** T-cell receptor (TCR) signaling genes influence the generation of Th2 cells and type 2 inflammation. **(C)** IEI associated with the IL-6-STAT3 cytokine signaling pathway.

Wiskott–Aldrich syndrome (WAS) is an X-linked recessive disease characterized by atopic dermatitis, thrombocytopenia, and combined immune deficiency (36). WAS results from mutations in the WAS protein (WASp), which is critical for actin polymerization affecting immunological synapse formation, mechanotransduction, and tissue migration in multiple hematopoietic cell lineages (37). WASp forms a complex with WASp-interacting protein (WIP) and dedicator of cytokinesis 8 (DOCK8), a guanine nucleotide exchange factor that is essential for WASp’s TCR-dependent function (38). In its GTP-bound form, the small Rho GTPase cell division cycle 42 (CDC42) binds and activates WASp (39). This causes a conformational change in the WASp C-terminus that activates the actin-related protein 2/3 (ARP2/3) complex (40) and leads to actin polymerization (**Figure 2A**). Less common bi-allelic LOF mutations in the genes encoding WIP (41, 42) and ARP2/3 subunit 1B (43, 44) have been described in individuals with similar clinical presentations to WAS, including elevated IgE and eosinophilia.

Autosomal recessive DOCK8 deficiency leads to a combined immunodeficiency with many shared features with WAS including atopic dermatitis, food allergies with increased incidence of anaphylaxis, recurrent viral infections, and an increased risk for autoimmunity and malignancies (45–48). CD4^+^ T cells from DOCK8-deficient patients are biased toward a Th2 cell fate (49). Studies in DOCK8-deficient mice have recently identified a novel CD4^+^ T-cell subset, Tfh13, which drives high-affinity IgE responses following allergen exposure and promotes anaphylaxis (50). WAS and DOCK8 deficiencies are of particular interest to understanding atopic diatheses given their diverse functions in TCR-dependent actin assembly and the formation of the immunological synapse (38), IL-2-dependent STAT5 phosphorylation and T regulatory cell function (51), and composition of the microbiome (52). Rare defects in serine/threonine-protein kinase 4 (*STK4*), which is upstream of DOCK8, have also demonstrated combined immunodeficiencies with many of the expanded features of WAS and DOCK8 deficiency including poor viral control, malignancy, autoimmunity, severe atopic dermatitis, eosinophilia, and elevated IgE (53, 54). Whether similar mechanisms underlie *STK4*’s pleiotropic effects require further investigation.

## Aberrant TCR Signaling

Human mutations affecting immunological synapse formation and TCR signaling molecules, resulting in aberrant or impaired TCR signal strength, have been associated with combined immunodeficiencies with severe atopic phenotypes (**Figure 2B**). The CARD11–BCL10–MALT1 (CBM) complex links T- and B-cell receptor engagement with the nuclear factor-kappa B (NF-κB) (55) and mTOR (56) signaling pathways. IEI involving the CBM complex can manifest in a broad range of clinical phenotypes. Complete LOF in Caspase Recruitment Domain Family Member 11 (*CARD11*, also called *CARMA1*) mutations result in severe combined immunodeficiency, while dominant negative mutations in *CARD11* can cause combined immunodeficiency and atopy (57–59). Autosomal dominant gain-of-function (GOF) mutations have also been described in individuals with “B cell expansion with NF-κB and T cell anergy” (BENTA) disease and B-cell malignancies, highlighting differences in regulation of antigen receptor signaling pathways in T and B cells (60). Two different cases of complete LOF mutations B cell CLL/lymphoma 10 (*BCL10*) (61, 62) have been described with a combined immunodeficiency without atopic features, though some patients with IEI involving mucosa-associated lymphoid tissue lymphoma translocation protein 1 (*MALT1*) paracaspase have presented with severe atopic dermatitis in infancy (63–65). Interestingly, in a genome-wide association study (GWAS) of the Learning Early About Peanut allergy (LEAP) study participants, the top gene associated with independent risk of developing peanut allergy in the peanut avoidance group was *MALT1* (66). While the extent to which the atopic phenotypes observed in LOF mutations in the CBM complex are explained by disrupting downstream NF-κB signaling is not clear, a retrospective analysis of patients with IEI registered in the USIDNET revealed that more than half of patients with NF-κB Essential Modulator (*NEMO*, also known as *IKBKG*) deficiency were noted to have atopic dermatitis (67). A more recent analysis of eosinophilia and elevated IgE in the USIDNET revealed that 3 out of 3 patients with *NFKB2* defects had at least one atopic manifestation and eosinophilia above the upper limit of normal in the reference population and an increased proportion of patients with *IKBKG* defects had both eosinophilia and elevated IgE (68).

Capping protein regulator and myosin 1 linker 2 (*CARMIL2*, also known as *RLTPR*) integrates TCR and CD28 co-stimulation signaling at the immunological synapse. *CARMIL2* deficiency results in variable clinical presentations, including a combined immunodeficiency with atopic dermatitis and eosinophilic esophagitis (69–72). Diffuse warts resulting from uncontrolled human papillomavirus infection is another feature reported in many of these patients. Interestingly, CD28 deficiency has recently been described in humans and revealed susceptibility to human papillomavirus infection with severe warts or giant cutaneous horns, though only 1 out of 3 patients were noted to have atopic features of food allergy and asthma (73). Whether this association is significant will require larger patient cohorts and future investigation; however, autoantibodies against CD28 have been associated with atopic disease (74).

Deficiency in the zeta chain of T-cell receptor associated protein kinase 70 (*ZAP70*) (75) resulting in impaired but not absent TCR signaling can present with significant atopic manifestations before an immunodeficiency is evident. Transgenic mice expressing a homologous point mutation found in humans in Linker for Activation of T cells (*LAT)* develop Th2 lymphoproliferation, systemic inflammation, and autoimmunity (76, 77). Additional studies in mice taking advantage of a variety of experimental approaches including TCR transgenic systems and altered peptide ligands to control for individual TCR affinities, varying antigen loads, and peptide:MHCII tetramer selection to remove high-affinity polyclonal CD4^+^ cells have further supported a model where weak TCR stimulation favors a Th2 differentiation program, whereas strong TCR stimulation can suppress GATA3 transcription and Th2 differentiation (78–81). Low TCR signal strength appears to promote Th2 cell induction independent of adjuvant effects (82). The intrinsic capacity of TCR signaling to influence T-cell fate decisions was further demonstrated with adoptive transfer of single naive T-cell clones, each expressing a unique TCR, into normal murine hosts and immunizing under identical conditions. Individual clones produced different patterns of effector T-cell and Tfh subsets, predicted by the strength of TCR signaling (83). Thus, numerous studies now support a dominant role of TCR signal strength in shaping helper T-cell differentiation and function *in vivo* (7).

## Disrupted Cytokine Signaling Pathways

Cytokines produced by innate cells following activation through pattern recognition receptors (PRRs) provide a third signal in addition to TCR and co-stimulatory molecules involved in T-cell activation and differentiation. LOF variants in cytokine and cytokine-induced signaling pathways that result in IEI with atopy reveal mechanisms that typically suppress Th2 in favor of other innate sensing-dependent responses (**Figure 2C**). IL-6 is an acute phase cytokine produced primarily by monocytes and macrophages with pleiotropic effects on hematopoietic and non-hematopoietic cell types. IL-6 binds its receptor (IL-6Rα) to form a hexameric complex with the co-receptor GP130 and activate the downstream JAK-STAT3 signaling pathway (84). In naïve T cells, this canonical pathway promotes the generation of Th17 and Th22 cells, which are important for clearance of extracellular infections. Supporting a simultaneous role of this pathway as a negative regulator of Th2 responses, two reports of different somatic LOF mutations in IL-6Rα in hematopoietic cells were recently identified in patients with atopic dermatitis and elevated IgE in addition to recurrent deep skin and lung infections (85, 86). IL-6R-deficient patients exhibit decreased STAT3 phosphorylation and increased frequencies of GATA3-expressing Th2 cells (85). LOF mutations in *IL6ST*, the gene encoding GP130, result in atopic dermatitis, elevated IgE, and thrombocytopenia, in addition to connective tissue, pulmonary, neurologic, and renal features (87), owing to the involvement of GP130 in multiple cytokine signaling pathways in numerous cell types (84). Autosomal dominant forms have been recently described with similar atopic features and increased Th2 cytokine production but less connective tissue defects, which may delineate IL-6- and IL-11-dependent GP130 functions (88).

Job’s syndrome was first described in 1966 in individuals with recurrent staphylococcal abscesses, pulmonary infections, and atopic dermatitis (89). This was later found to also be associated with elevated IgE and eosinophilia due to *STAT3* mutations with an autosomal dominant (AD) inheritance pattern (90), subsequently referred to as autosomal dominant hyper IgE syndrome (AD-HIES). STAT3 phosphorylation leads to its homodimerization, translocation to the nucleus, and transcription of downstream genes, including cytokines promoting Th17 differentiation and repression of Th2 cells. One such transcription factor, *ZNF341*, is involved in basal and inducible *STAT3* gene expression and patients with *ZNF341* mutations have been described with a similar phenotype to AD-HIES (91, 92). STAT3 LOF also have reduced anaphylaxis responses despite highly elevated IgE levels, owing to effects of *STAT3* influencing mast cell activation in response to IgE (93) as well as endothelial cell responsiveness to histamine (94).

Descriptions of monogenic IEI are also supported by GWAS studies that have identified IL-6R polymorphisms associated with atopic dermatitis, elevated IgE, and asthma (95–97). Interestingly, a patient with neutralizing anti-IL-6 autoantibodies was described with Staphylococcal skin infections similar to those that affect patients with atopic dermatitis, but otherwise lacked an atopic phenotype (98). Similarly, the treatment of various inflammatory diseases with anti-IL-6 monoclonal antibodies has not resulted in reports of significantly increased atopic manifestations (99). This reinforces that the timing of immune development is critical for the pathogenesis of atopic disease, perhaps through interactions with the microbiome or “early life imprinting” (100).

## Decreased T-Cell Repertoire Diversity

**Figure 3:**
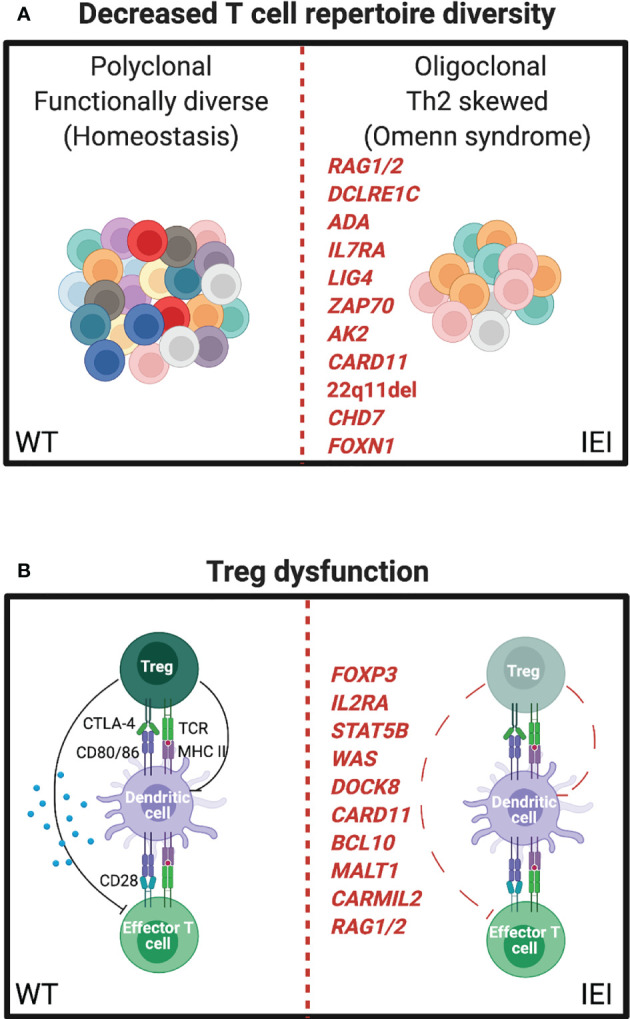
IEI influencing the T-cell repertoire can manifest as severe atopy. **(A)** Left, resting polyclonal T cells in a normal (WT) immune repertoire. Right, oligoclonal expansion of T cells in leaky SCID, often leading to Th2 skewing and eosinophilia (Omenn syndrome). **(B)** Left, normal balance between regulatory T cell (Treg) and effector T cell (Teff) functions. Right, mutations affecting Treg number or function leading to autoimmune and atopic phenotypes.

Severe combined immunodeficiency (SCID) is defined by very low or absent T-cell number and function. Prior to the addition of T-cell receptor excision circles to the newborn screening program, patients with SCID typically presented in the first year of life with failure to thrive and life-threatening infections. Immune dysregulation and atopy are not features of classical SCID, owing to the lack of T cells necessary to cause immunopathology. However, it is now appreciated that there is a spectrum of SCID and combined immunodeficiency that includes at least 64 distinct genetic disorders (101). Along this spectrum, whereas null mutations in many of these genes result in SCID, numerous hypomorphic variants with partial function have now been described and lead to “leaky SCID” and Omenn syndrome. In addition to significantly increased risk of infections including opportunistic infections, individuals with Omenn syndrome develop a lymphoproliferative state with clonal expansion of a limited number of T cell clones that lead to damaging tissue infiltration of the skin and other organs (**Figure 3A**). Oligoclonal T-cell expansion in Omenn syndrome is associated with Th2 differentiation, secretion of type 2 cytokines, elevated total serum IgE, and eosinophilia (102). Histologically, the erythrodermic skin inflammation of Omenn syndrome very closely resembles that of severe atopic dermatitis.

Omenn syndrome has been well described for a number of genes including recombination activating gene 1 and 2 (*RAG1/RAG2*) (103), ARTEMIS (*DCLRE1C*) (104), adenosine deaminase (*ADA*) (105, 106), IL-7 receptor alpha (*IL7RA*) (107), DNA ligase 4 (*LIG4*) (108), *ZAP70* (75), adenylate kinase 2 (*AK2*) (109), and *CARD11* (110). Thymic defects, in DiGeorge syndrome (22q11del) (111), CHARGE syndrome (*CHD7*) (112), and forkhead box protein N1 (*FOXN1*) (113), can also present with Omenn syndrome. While it is not entirely clear how these heterogenous disorders lead to the common atopic features associated with Omenn syndrome, lymphopenia-induced homeostatic proliferation, insufficient thymic deletion of autoreactive T-cell clones, insufficient Treg generation, uncontrolled viral infections, and stochastic variability in effector T-cell differentiation are possibilities.

Murine studies have shed further light on the impact of T-cell repertoire diversity. Milner and Paul et al. reconstituted lymphopenic *RAG-/-* mice with different numbers of polyclonal naïve CD4^+^ T cells from a C57BL/6 host with normal thymic development to demonstrate that a limited repertoire results in preferential Th2 skewing, IgE production, and multiorgan eosinophilic inflammation when compared to more complete immune repertoire reconstitution (114). Importantly, tissue pathology could be prevented by altering the balance of Tregs to conventional CD4^+^ T cells in this system, and IEI associated with Treg dysfunction provide further evidence for their role in limiting atopic disease.

## Altered Balance of Conventional T cells and Regulatory T Cells

Inborn errors specifically associated with Treg dysfunction have clarified their important role in autoimmunity, atopy, and tissue homeostasis. Tregs are dependent on the expression of the master transcription factor forkhead box protein P3 (FoxP3). Early descriptions of FoxP3 deficiency in humans resulted in “X-linked autoimmunity-allergic dysregulation syndrome” and “Immunodysregulation polyendocrinopathy enteropathy X-linked” (IPEX) syndrome (115–117). Features of IPEX include multisystem autoimmunity and severe atopy including eczema, food allergy, and eosinophilic inflammation. FoxP3 deficiency in *scurfy* mice recapitulates many atopic and autoimmune features of IPEX in humans (118), and Treg-specific disruption of multiple immune pathways leads to systemic type 2 inflammation (119–122).

IL2RA, the high-affinity IL-2 receptor, is most highly expressed on Tregs and is critical for their survival and function through the actions of the transcription factor *STAT5B*. *IL2RA*- and *STAT5B*-deficient patients can also exhibit a combined immunodeficiency with autoimmunity, atopic dermatitis, and elevated IgE (123, 124) in addition to non-hematopoietic effects on growth found in *STAT5B* deficiency. Further evidence supporting a central role for Tregs as regulators of Th2 immunity and atopic disease is the multiple reports of IEI described above also affecting Treg diversity or function, including *WAS* (125–128), *DOCK8* (45, 51), CBM complex components (129–131), *CARMIL2* (132), and *RAG* (133) (**Figure 3B**).

## Innate Cell Effector Mechanisms

Monogenic disorders primarily affecting innate immune cells and manifesting with unique atopic features have also been described. The PLCγ2-associated antibody deficiency and immune dysregulation (PLAID) results from temperature-sensitive spontaneous enzyme activity of PLCγ2 leading to mast cell degranulation. Individuals with PLAID have cold or evaporative cooling-induced urticaria (134–136). The cold-induced urticaria in individuals with PLAID can trigger burning sensations in the mouth following consumption of cold foods, which mimics symptoms of food allergy, but does not lead to anaphylaxis. Syncopal episodes have been described following prolonged exposures to cold swimming pools, however.

## Summary

Here, we discussed human monogenic disorders associated with significant type 2 inflammation and atopic disease. This review is not a comprehensive list of all IEI associated with increased risk of atopic manifestations, but rather an attempt to illustrate key mechanistic pathways that drive unwanted type 2 immune responses in humans and are supported by experimental mouse models. Impaired barrier function or dysregulated Th2 responses are central drivers of common atopic disease and IEI offer considerable insight into disease pathogenesis (summarized in **Table 1**). Further awareness of the features and mechanisms of IEI will aid in improved diagnosis and inform development of more targeted therapeutics for atopic disease.

**Table 1:** Monogenic IEI associated with atopic disease.

Gene	Inheritance	Immune Manifestations	Atopic Manifestations
**Disorders of barrier function**			
*FLG*	AR	Skin infections	A.D., FA, A.R., As, eos, high IgE
*SPINK5*	AR	Skin infections	A.D., FA, A.R., As, eos, high IgE
*CDSN*	AR	Skin infections	A.D., FA, eos, high IgE
*DSG1*	AR	Skin infections	A.D., FA, eos, high IgE
*DSP*	AR	Skin infections	A.D., FA, eos, high IgE
**Cytoskeletal abnormalities**			
*WAS*	XL	CID	A.D., FA, eos, high IgE
*WIP*	AR	CID	A.D., FA, eos, high IgE
*DOCK8*	AR	CID	A.D., FA, eos, high IgE
*STK4*	AR	CID	A.D., FA, eos, high IgE
**Aberrant TCR signaling**			
*CARD11*	AR	CID, SCID	Eos, high IgE
*BCL10*	AR	CID, SCID	Eos, high IgE
*MALT1*	AR	CID, SCID	Eos, high IgE
*CARMIL2*	AR	CID	Eos, high IgE
*ZAP70*	AR	CID, SCID	Eos, high IgE
*LAT*	AR	CID, SCID	Eos, high IgE
**Cytokine pathways**			
*IL6RA*	AR	Skin, lung infections	A.D., eos, high IgE
*IL6ST*	AR, AD	Skin, lung infections	A.D., eos, high IgE
*STAT3*	AD	Skin, lung infections	A.D., eos, high IgE
*ZNF341*	AR	Skin, lung infections	A.D., eos, high IgE
**Decreased T-cell repertoire**			
Multiple genes presenting as Omenn syndrome	AR	Leaky SCID	Erythroderma, eos, high IgE
**Regulatory T-cell defect**			
*FOXP3*	XL	AI	A.D., FA, As, eos, high IgE
*IL2RA*	AR	CID, AI	A.D., FA, As, eos, high IgE
**Innate cell effector mechanisms**			
*PLCG2*	AD	CVID, AI, autoinflammatory	Temperature-sensitive mast cell degranulation

A summary of causative genes, heritability, and major clinical features is presented. Table includes disorders of barrier function where infections and atopy overlap with IEI. Overlapping mechanisms are discussed further in the main text. AD, autosomal dominant; AR, autosomal recessive; XL, x-linked; CID, combined immune deficiency; SCID, severe combined immune deficiency; CVID, common variable immune deficiency; A.D., atopic dermatitis; eos, eosinophilia; AI, autoimmunity; A.R., allergic rhinitis; FA, food allergy; As, asthma.

## Author Contributions

All authors listed have made a substantial, direct, and intellectual contribution to the work and approved it for publication.

## Funding

This work was supported by the National Institutes of Health grant NIAID T32 AI007512 (RWN) and AI139633 (RSG). Figures created with BioRender.com.

## Conflict of Interest

The authors declare that the research was conducted in the absence of any commercial or financial relationships that could be construed as a potential conflict of interest.

## Publisher’s Note

All claims expressed in this article are solely those of the authors and do not necessarily represent those of their affiliated organizations, or those of the publisher, the editors and the reviewers. Any product that may be evaluated in this article, or claim that may be made by its manufacturer, is not guaranteed or endorsed by the publisher.
